# Integration of PARP-inhibitors in ovarian cancer therapy

**DOI:** 10.37349/etat.2020.00011

**Published:** 2020-06-29

**Authors:** Antonella Pietragalla, Francesca Ciccarone, Camilla Nero, Giovanni Scambia, Domenica Lorusso, Gennaro Daniele

**Affiliations:** 1 Scientific Directorate, Fondazione Policlinico Universitario A. Gemelli IRCCS, 00168 Rome, Italy; 2 Catholic University of the Sacred Heart, 00168 Rome, Italy; University of Southampton, UK

**Keywords:** PARP-inhibitors, ovarian cancer, *BRCA1* and *2*, homologous recombination deficiency, DNA repair

## Abstract

Poly-ADP-ribose polymerase inhibitors (PARP-I) represent one of the most attractive and promising class of biological agents studied both in relapsed ovarian cancer (OC) and in the advanced setting. The availability of this new class of drugs has changed the clinical management of OC ensuring an unprecedented advance in such an aggressive cancer. Three oral PARP-I are currently available: olaparib, niraparib and rucaparib. Another two are in active clinical exploration: veliparib and talazoparib. Here the authors report clinical data with PARP-I with a particular emphasis on the phase II and III trials that support PARP-I approval by regulatory agencies in OC patients.

## PARP-I development

### Mechanisms of DNA repair

Five different mechanisms support, with different degrees of fidelity, genomic stability during the cell cycle: base excision repair (BER), nucleotide excision repair (NER), mismatch repair (MMR), homologous recombination (HR) and non-homologous end joining repair (NHEJ) [1]. Poly-ADP-ribose polymerase (PARP) family proteins encompasses 17 different nuclear enzymes but only PARP-1, PARP-2 and PARP-3 have a recognized role in the DNA repair machinery with PARP 1 as the most frequent isoform. PARP acts in several DNA repair mechanisms but is most extensively described in BER where it promotes the formation of DNA repair complexes [2]. However, PARP1 is known to play a crucial role also in HR facilitating the process throughout all the phases [3].

**Figure 1.**
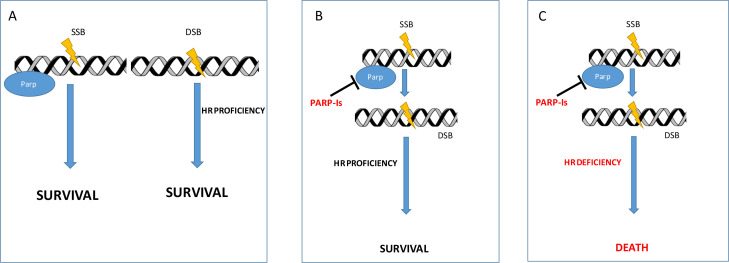
Synthetic lethality. (A) Normal cell with all the DNA repair mechanisms functioning; (B) PARP inhibition determines SSB accumulation that are effectively repaired in HR proficient cells; (C) PARP inhibition determines SSB accumulation that ultimately ends in multiple DSB cannot be repaired by HR deficient cells (synthetic lethality). DSB: double-strand break; SSB: single-strand breaks

The development of PARP inhibitors (PARP-I) stems from the observation that these molecules were able to selectively kill cancer cells defective of *BRCA* 1 and 2 tumour suppressors [4, 5]. This capability resides on the mechanism that thereafter is called “synthetic lethality” (Figure 1). Based on this model, the absence of either one of the DNA repair activities is not lethal for the cell. But combining the deficiency of BRCA-mediated mechanism of repair (HR) with inhibition of PARP becomes highly lethal. Furthermore, other proteins have been related to the potential increased sensitivity to PARP-I, but their role in the clinical setting is still unclear [6]. Since the observation that BRCA defective tumours derive a great benefit from PARP treatment [7], ovarian cancer (OC) has been the privileged ground of development of these agents.

## PARP-I for recurrent disease

PARP-I efficacy and safety have been extensively investigated in relapsed OC patients. Three oral PARP-I, olaparib, niraparib and rucaparib, currently received authorization by the Food and Drug Administration (FDA) and European Medicines Agency (EMA) both in relapsed OC as maintenance therapy after response to platinum-containing treatment and as monotherapy treatment of *BRCA1/2* mutated relapsed OC. Here we report the phase II and III trials that support PARP-I approval by regulatory agencies in relapsed OC patients.

### Olaparib

Initially, the olaparib authorization by the FDA and EMA was carried out with two different indications. In 2014 the FDA approved olaparib as monotherapy in germline *BRCA1/2* (gBRCA) mutated advanced OC patients treated with more than three previous lines of chemotherapy (Study 42, NCT01078662). In the same period, EMA authorized olaparib (capsules formulation) as maintenance treatment in platinum-sensitive relapsed *BRCA1/2* mutated high-grade serous OC (HGSOC) (Study 19, NCT00753545). Subsequently, both the FDA and EMA extended approved olaparib indication (tablet formulation) in the maintenance setting regardless of *BRCA1/2* mutation (SOLO2 trial, NCT01874353).

Study 42 is a large single-arm, phase II study that evaluated olaparib capsule treatment (400 mg *BID*) in terms of response rate in *BRCA1/2*-mutated recurrent cancers (ovarian, breast, prostate, and pancreatic).

One hundred and ninety-three OC patients, heavily pre-treated, (4.3 median number of chemotherapy lines for advanced disease) were included [8]. The objective response rate was 34%. Recently, data from a subgroup of *BRCA1/2* mutated OC patients (more than 3 prior chemotherapy regimens) showed a 34% overall response rate and a median response duration of 7.9 months [9].

Subsequently, the phase III trial SOLO3 (NCT02282020), enrolled 266 g*BRCA* mutated platinum sensitive relapsed OC patients to olaparib tablets (300 mg *BID*) or standard treatment (weekly paclitaxel, weekly topotecan, gemcitabine or pegylated liposomal doxorubicin). The trial confirmed olaparib efficacy and safety profile with an overall response rate (ORR) of 72% *vs.* 51% with chemotherapy (OR 2.53, 95% CI 1.40–4.58, *P* = 0.002). The most frequent adverse events (AEs) reported during olaparib treatment were nausea (65%) and anemia (50%). Regarding AEs grade ≥ 3 experienced, anemia (21% olaparib *vs.* 0% chemotherapy), palmar-plantar erythrodysesthesia (0% olaparib *vs.* 12% chemotherapy) and neutropenia (6% *vs.* 11%) were reported [10].

In relapsed platinum sensitive OC maintenance setting, olaparib has been evaluated in two international prospective (Study 19 and SOLO2/ENGOT-Ov21) trials [11].

Study 19 is a randomized phase II trial. Two hundred sixty-five patients with relapsed high grade serous OC, in complete (CR) or partial response (PR) to platinum-containing chemotherapy were treated with olaparib (*n* = 136) or placebo (*n* = 129) 400 mg (capsules formulation) twice daily. Olaparib treatment shows a significantly longer median progression-free survival (PFS) compared with placebo (8.4 *vs.* 4.8 months, HR 0.35, *P* < 0.001) [11]. In the *BRCA1/2* (somatic and germline) mutated patients the median PFS was 11.2 *vs.* 4.3 months for placebo (HR 0.18, *P* < 0.001), as shown in a study 19 pre-planned subgroup analysis (136 patients) [12].

No significant difference in terms of overall survival (OS) was observed in overall population and in *BRCA* mutated patients: intention-to-treat (ITT) population OS was 29.8 *vs.* 27.8 months; (HR 0.73, *P* = 0.025), in *BRCA1/2* mutated patients OS was 34.9 *vs.* 30.2 months; (HR 0.62, *P* = 0.025) [13]. AEs olaparib related were nausea, fatigue, vomiting, and anemia, which were mostly of grade 1 or 2 and arose within the first 4–8 weeks of treatment. No significant detrimental effects in terms of quality of life were observed.

Subsequently, SOLO2/ENGOT-Ov21 trial confirmed the Study 19 results with a new olaparib formulation (tablet *vs.* capsules). SOLO2/ENGOT-Ov21 is a phase III trial designed to assess efficacy and safety of olaparib 300 mg twice daily *vs.* placebo in *BRCA1/2* mutated patients in response to platinum-containing chemotherapy. Stratification criteria were response to previous platinum chemotherapy (complete or partial) and platinum-free interval (> 6–12 months or > 12 months). Olaparib treatment showed an impressive and significant PFS when compared to placebo (19.1 *vs.* 5.5 months, HR 0.30, *P* < 0.0001).

Nausea, fatigue, vomiting, and anemia, primarily of grade 1 or 2, were the AEs more frequently reported in olaparib arm [14].

On August 2017 and May 2018, the FDA and EMA respectively extended olaparib indication as maintenance treatment of platinum sensitive relapsed OC patient, in response to last platinum-based chemotherapy, irrespective of *BRCA* status.

### Niraparib

Niraparib is a selective inhibitor of PARP-1 and PARP-2 enzymes. The FDA and EMA niraparib approval (March and November 2017 respectively) comes from the ENGOTOV16/NOVA trial.

ENGOT OV16/NOVA trial is a phase III, double-blinded, trial that enrolled relapsed platinum sensitive OC patients treated with at least two previous platinum-based regimens.

The trial includes patients with measurable disease less than 2 cm treated within 8 weeks of last platinum chemotherapy regimen. Two hundred and three *BRCA* mutated and 350 non-g*BRCA* mutated patients assessed with Myriad BRCAnalysis test, were randomized 2:1 to niraparib 300 mg/day or placebo. The primary outcome of the study was PFS.

The PFS was 21 *vs.* 5.5 months (HR 0.27, *P* < 0.0001) in the *BRCA* mutated population, while in the wild type g*BRCA* patient PFS was 9.3 *vs.* 3.9 months (HR 0.45, *P* < 0.001). In homologous recombination deficiency (HRD)-positive cohort, according to Myriad Genetics myChoice HRD test, PFS was 12.9 *vs.* 3.8 months (HR 0.38, *P* < 0.001) [15].

Functional Assessment of Cancer Therapy-Ovarian Symptom Index reveals no significant detrimental effects [16]. We are still awaiting OS data, but the estimated analysis of PFS at 24 months in the niraparib arm was 0.42 (0.30, 0.55) *vs.* 0.16 (0.07, 0.28) (g*BRCA*mut) and 0.27 (0.19, 0.35) *vs.* 0.12 (0.06, 0.21) (non-g*BRCA*mut) [17].

Niraparib’s AE are similar to the PARP-I class. However, grade 3–4 thrombocytopenia, is reported in 34% of patients and typically appears during the first three cycles of therapy. Other grade G3-G4 AE reported to treatment are anemia (25%) and neutropenia (20%). If a dose reduction is correctly performed, only 3%, 2%, and 1% of patient will stop niraparib treatment due to thrombocytopenia, neutropenia, and anemia.

Body weight < 77 kg or basal platelet count < 150, 000 μL are risk factors to develop grade 3 thrombocytopenia (exploratory analysis): in this setting of patient the start dose of niraparib at 200 mg/day when one or both risk factors are present did not impact on PFS [18].

Regarding monotherapy treatment, the single arm Quadra trial (NCT02354586) results are available [19]. The study is a single arm trial evaluating niraparib efficacy in 45 platinum sensitive, HRD-positive relapsed OC patients treated with or more than three previous chemotherapy lines and PARP-I treatment naive. The trial shows an ORR of 27.5% and a disease control rate of 68.6% with duration of response of 9.2 months [19]. Quadra trial supports the recent (October 2019) FDA approval for niraparib in advanced HRD-positive OC patients treated with more than 3 previous chemotherapy regimens.

### Rucaparib

Rucaparib is a potent PARP-1, 2 and 3 inhibitor approved by FDA and EMA for the treatment of patients with *BRCA1/2* mutated (germline or somatic) OC treated with two or more lines of previous chemotherapy [20].

Data about rucaparib efficacy for this setting are drawn from Study 10 and ARIEL2 (NCT01891344) clinical trials. [21, 22].

Study 10 is a three-part phase I/II clinical trial: part 1 determined as 600 mg twice daily the maximum tolerable dose (MTD) and assessed the drug pharmacokinetics. In the phase II, 42 platinum sensitive (platinum free interval more than 6 months) patients with high-grade OC already treated with two to four previous regimens were enrolled. The investigator-assessed ORR was 59.5%, with a median duration of response of 7.8 months (95% CI 5.6–10.5) [21].

According to encouraging results, ARIEL2 trial was conducted [22].

The ARIEL2 study is a multicenter, phase II open label trial evaluating the activity of rucaparib as single agent in relapsed HGSOC or high-grade endometrioid ovarian carcinoma (HGEOC) regardless *BRCA* mutation status in patients treated with at least one prior platinum containing regimen. Two hundred and four patients (*n* = 40 *BRCA* mutated, 82 *BRCA* wild type/LOH high, *n* = 70 *BRCA* wild type/LOH low, *n* = 12 wild type/LOH unclassified) were enrolled.

According to HRD, patients were classified into three subgroups: *BRCA* mutated (pathogenetic germline or somatic), *BRCA* wild-type and LOH high group, and *BRCA* wild-type and LOH low group. The biomarker chosen for HRD was genomic LOH, with a cutoff to define LOH high of ≥ 14%.

The study reported a median PFS of 12.8 months (95% CI 9.0–14.7) for *BRCA* mutated group, in contrast to 5.7 months (95% CI 5.3–7.6) and 5.2 months (95% CI 3.6–5.5) in the LOH high and low groups, respectively. This study allowed the optimization of LOH degree associated with clinical efficacy that was prospectively defined for ARIEL3 phase III trial and Part 2 of ARIEL2 as ≥ 16%.

Subsequently, a pooled analysis from 106 patients (*n* = 42 from Study 10 Part 2A, *n* = 64 from ARIEL2 Parts 1 and 2) *BRCA* mutated high-grade relapsed OC after two or more chemotherapy lines treated with rucaparib at a starting dose of 600 mg twice daily was performed. Patients were already treated with a median of three prior lines of chemotherapy (range, 2–6), and 43% of patients had received at least three prior platinum-containing therapies. The ORR was 53.8% (95% CI 43.8–63.5) with 8.5% and 45.3% of patients in CR and PR, respectively. The median duration of response was 9.2 months (95% CI 6.6–11.6).

Grade ≥ 3 anemia was reported in 24.9% of patients. Treatment interruption, dose reduction, and treatment discontinuation related to AEs was reported in 58.6%, 45.9%, and 9.8% of patients, respectively [23].

Rucaparib received the FDA (April 2018) and EMA (January 2019) approval as maintenance treatment for recurrent epithelial OC, achieving a CR or PR to platinum-containing chemotherapy according to ARIEL3 trial (NCT01968213) results.

In this study, 564 patients previously treated with more than 2 prior lines of chemotherapy were randomized 2:1 to receive rucaparib or placebo maintenance (600 mg oral *BID*). Enrollment was not dependent on BRCA molecular analysis, patients were stratified into 3 groups: a *BRCA*-mutant group, an HRD-positive group, and an intent-to-treat group (all patients).

A median PFS of 16.6 months in the rucaparib arm and 5.4 months in the placebo arm (HR 0.23, 95% CI 0.16–0.34, *P* < 0.0001) for *BRCA*-mutated patients was reported; in the HRD-positive population PFS was 13.6 *vs.* 5.4 months (HR 0.32, 95% CI 0.24–0.42, *P* < 0.0001) while in the overall population (ITT) patients, 10.8 and 5.4 months respectively (HR 0.36, 95% CI 0.30–0.45, *P* < 0.0001) [24].

ARIEL3 trial confirmed the efficacy of PARP-I, regardless of *BRCA* mutations or the HRD status. Grade 3/4 AEs were reported in 56% for the rucaparib arm *vs.* 15% in the placebo arm. The most common reported AEs were anemia (19% *vs.* 1%) and an increased alanine or aspartate aminotransferase (10% *vs.* 0%). To assess the HRD status, the FDA simultaneously approved, FoundationFocus™ CDx *BRCA* LOH as companion diagnostic test [25].

Finally, ARIEL 4 (NCT02855944) is an ongoing phase III study evaluating the rucaparib activity (600 mg *BID*) *vs.* standard chemotherapy (weekly paclitaxel or platinum-containing regimen for platinum free interval < 12 months and > 12 months respectively) in *BRCA* mutated OC patients treated with more than two previous lines.

### Veliparib

Veliparib is a small oral PARP-I with selective activity for PARP1 and PARP2 with a weaker trapping compared with other PARP-I. A phase I/II trial (NCT01472783) involving g*BRCA1/2* mutated relapsed OC patient, established the MTD as 300 mg twice daily with an ORR of 65%.

Veliparib efficacy and tolerability as a single agent (400 mg twice daily) has been evaluated in the phase II clinical trial Gynecologic Oncology Group-0280 (NCT01540565). The study enrolled 45 relapsed *BRCA1/2* mutated OC patients pretreated with no more than three prior chemotherapy regimens. The study reported a median PFS of 8.1 months (0.43–19.55 months) and median OS was 19.7 months with an acceptable toxicity profile [26].

### Talazoparib

Talazoparib is another potent PARP1-2 inhibitor (PARP1 IC50: 0.57 nM), with a proven effectiveness direct to *BRCA1/2 in vitro* and *in vivo* models. The clinical experience of Talazoparib in OC is very limited.

A phase I/II trial (NCT01286987), conducted in 113 patients with advanced solid tumors reported an acceptable safety profile as single-agent (1.0 mg/day). In OC cohort an ORR of 42% (5/12 patients) [27] was observed.

Anemia (23%), thrombocytopenia (18%), and neutropenia (10%) were the most commonly grade 3–4 AEs reported.

In platinum-resistant OC, the combination of talazoparib with temozolomide or irinotecan demonstrated PRs in 4/7 (57%) [28].

A phase phase I/II trial of talazoparib in association with carboplatin in patients with solid tumors demonstrated a 52% of disease stabilization (range, 7–22 weeks; median, 10.5 weeks) [29].

## Front line

In 2018, the results of the SOLO-1 trial investigating olaparib in maintenance after front-line platinum based chemotherapy were published [30]. In this trial, 391, *BRCA1/2* mutated (deleterious or suspected deleterious germline or somatic mutation), FIGO III-IV, high grade OC patients were randomised (2:1) to receive either olaparib or placebo (300 mg *BID* or placebo for 2 years) as maintenance after partial or complete response to front-line platinum based chemotherapy. The study showed an unprecedented result in term of the primary outcome (PFS). In particular, the Kaplan-Meier estimate of the risk of being progression/death free at 3 years was 60% for patients receiving olaparib *vs.* 27% for those receiving placebo (HR = 0.030, 95% CI 0.23–0.41) [30]. Unfortunately, at the time of the results presentation the data on overall survival were not mature and we do not have the mature data yet. Based on these data the FDA and EMA approved olaparib as maintenance therapy for all the *BRCA1/2* mutated OC patients, experiencing an objective response after a front-line platinum-based treatment.

More recently, the PAOLA-1 trial [31] tested the hypothesis that olaparib added in maintenance to bevacuizumab improves the PFS as compared with bevacizumab alone, in patients who received bevacizumab as part of the front-line treatment.

In this investigator initiated trial, were enrolled 762 patients who received at least 3 bevacizumab courses along with the platinum based chemotherapy. Although patients were allowed to participate irrespective of the *BRCA* status, *BRCA* mutation has been addressed for all the patients and HRD (Myriad MyChoice ≥ 42) for the vast majority (82%) of the total. Patients with high grade serous or endometroid OC treated with carboplatin-paclitaxel-bevacizumab chemotherapy in partial or complete response, were randomized (2:1) to continue bevacizumab alone (15 mg/kg every 3 weeks on day 1) or bevacizumab in combination with olaparib (300 mg twice daily for up to 24 months) for 24 months.

Main analysis showed a significant benefit with olaparib (22.1 *vs.* 16.6 months, HR = 0.59, 95% CI 0.49–0.72) in the overall population. Also, in this case the maximum benefit was observed in the *BRCA* mutant population (HR = 0.31, 95% CI 0.20–0.47) and in the HRD patients (HR = 0.33, 95% CI 0.25-0.45) with the median PFS reaching 37 months.

Interestingly, the safety data showed a reasonable profile for the combination, although with an increased incidence of AEs in the experimental arm: grade ≥ 3 AEs occurred in 57% *vs.* 51% of patients receiving olaparib/bevacizumab and placebo/bevacizumab, respectively, most commonly hypertension (19% *vs.* 30%) and anemia (17% *vs.* < 1%).

In the PRIMA study, 733 patients with either serous or endometroid OC with newly diagnosed stage III-IV high-grade serous OC were randomised to receive either niraparib or placebo (300 mg/once daily) for 36 months whether they had an objective response to first-line chemotherapy. Interestingly, the patients were allowed into the trial irrespective of the *BRCA* status, although they were all tested for HRD (through Myriad MychoiceHRD®) [32]. In fact, the primary endpoint of the study, the PFS, was tested hierarchically first in the HRD population and, if statistically significant into the whole population (ITT analysis). Among the HRD patients, (i.e. MyChoice ≥ 42 or *BRCA* mutant) niraparib yielded a median PFS of 21.9 as compared with the 10.9 months with placebo (HR = 0.43, 95% CI 0.31–0.59). The ITT analysis confirmed an improvement of PFS with niraparib (13.8 *vs.* 8.2 months, HR = 0.62, 95% CI 0.50–0.76). The most commonly reported grade ≥ 3 AEs were anaemia in 31% of patients, thrombocytopenia in 29%, and neutropenia in 13% of patients overall.

The VELIA trial evaluated the potential role of veliparib combined with chemotherapy and given as maintenance as compared with platinum based chemotherapy alone [33]. In this trial 1, 140 patients, with newly diagnosed stage III-IV high-grade serous OC were randomised (1:1:1) to receive platinum-based chemotherapy alone, chemotherapy plus veliparib for 6 cycles, or chemotherapy plus veliparib followed by veliparib maintenance (veliparib throughout). In this trial *BRCA* and HRD assessment were done for all the patients. HRD status initially was added as strata in the randomisation after the half of the patients enrolled. For the definition of the HRD status, Myriad MychoiceHRD® > 33 threshold was used. The main comparison of the trial was between the arm with veliparib throughout and the chemotherapy alone. Also in this case, the primary outcome of the trial, the PFS, was first assessed in the *BRCA* mutant population then in the HRD population and, finally, in the ITT population. The results showed an increase of the PFS in all the 3 populations. In particular, the median PFS was 34.7, 31.9 and 23.5 months for the veliparib throughout arm as compared with 22 (HR 0.44, 95% CI 0.28–0.68), 20.5 (HR = 0.57, 95% CI 0.43–0.76) and 17.3 (HR = 0.68, 95% CI 0.56–0.83) months for the chemotherapy arm in the *BRCA* mutant, HRD and ITT populations, respectively.

Interestingly, although significantly more patients experienced toxic events in the veliparib arm, dose intensities delivered were not dissimilar among the arms, demonstrating that the combination is feasible [33]. Table 1 summarizes clinical trials results for PARP-I in ovarian cancer.

**Table 1.** Clinical trials results for PARP Inhibitors in ovarian cancer

**Study**	**Phase**	**Patients (*n*)**	**Setting**	**Treatment arms**	**Results**	***P*-value**
**Recurrent/relapsed setting**						
STUDY 42 [8]	II	193	Recurrent pre-treaed advanced OC, *BRCA*mut	Olaparib 400 mg *BID* (capsules)	ORR: 34% MDR: 7.9 months	
STUDY 19 [11] [12]	II	265	Platinum-sensitive recurrent HGSOC	Olaparib 400 mg *BID* (capsules) Placebo	Overall population: 8.4 *vs.* 4.8 months	*P* < 0.0001
					*BRCA*mut: 11.2 *vs.* 4.3 months	*P* < 0.0001
SOLO 2 [14]	III	295	Platinum-sensitive recurrent HGSOC or HGEOC, *BRCA*mut	Olaparib 300 mg *BID* Placebo	Median PFS	
					19.1 *vs.* 5.5 months	*P* < 0.0001
SOLO 3 [10]	III	266	Platinum-sensitive recurrent HGSOC *BRCA*mut	Olaparib 300 mg *BID* Single-agent nonplatinum chemotherapy (weekly paclitaxel, weekly topotecan, gemcitabine or pegylated liposomal doxorubicin)	ORR: 72.2% *vs.* 51.4	*P* = 0.002
NOVA [15]	III	553	Platinum-sensitive recurrent HGSOC	Niraparib 300 mg Placebo	Median PFS	
					g*BRCA*mut: 21 *vs.* 5.5 months	*P* < 0.001
					*BRCA*wt HRD^+^: 12.9 *vs.* 3.8 months	*P* < 0.001
					Overall non-g*BRCA*: 9.3 *vs.* 3.9 months	*P* < 0.001
QUADRA [19]	II	45	Platinum sensitive HRD positive HGSOC	Niraparib 300 mg	ORR: 27.5% DCR: 68.6% DoR: 9.2 months	
STUDY 10 [21]	I/II	42	Platinum-sensitive recurrent HGSOC or HGEOC, g*BRCA*mut (phase II PART 2A)	Rucaparib 600 mg *BID*	ORR: 59.5% MDR: 7.8 months	
ARIEL 2 PART 1 [22]	II	192	Platinum sensitive recurrent HGSOC or HGEOC	Rucaparib 600 mg *BID*	Median PFS	
					*BRCA*mut: 12.8 months	*P* < 0.0001
					*BRCA*wt LOH High: 5.7 months	*P* = 0.011
					*BRCA*wt LOH low: 5.2 months	*P* = 0.011
ARIEL 3 [24]	III	564	Platinum-sensitive recurrent HGSOC or HGEOC	Rucaparib 600 MG *BID* Placebo	Median PFS	
					*BRCA*mut: 16.6 *vs.* 5.4 months	*P* < 0.0001
					HRD^+^: 13.6 *vs.* 5.4 months	*P* < 0.0001
					ITTP: 10.8 *vs*. 5.4 months	*P* < 0.0001
**Front-line**						
SOLO1 [30]	III	391	FIGO Stage III/IV HGSOC or HGEOC *BRCA*mut	Olaparib 300 mg *BID* Placebo	*BRCA*mut: > 36 (NR) *vs.* 13.8 months	*P* < 0.0001 HR: 0.30 (95% CI 0.23–0.41)
PAOLA-1 [31]	III	806	FIGO Stage III/IV HGSOC or HGEOC	Bevacizumab: 15 mg/kg, q21 × 15 months, including when administered with chemotherapy + olaparib (300 mg *BID*) × 24 months Bevacizumab: 15 mg/kg, q21 × 15 months, including when administered with chemotherapy + Placebo × 24 months	Overall population ITT: 22.1 *vs.* 16.6 months	*P* < 0.0001 HR: 0.59 (95% CI 0.49–0.72)
					*BRCA*mut: 37.2 *vs.* 21.7 months	HR: 0.31 (95% CI 0.20–0.47)
					HRD (including *BRCA*): 37.2 months *vs*. 17.7 months	HR: 0.33 (95% CI 0.25–0.45)
					HRD (*BRCA*wt): 28.1 months *vs*. 16.6 months	HR: 0.43 (95% CI 0.28–0.66)
					HRP (*BRCA*wt): 16.9 months *vs*. 16.0	HR: 0.92 (95% CI 0.72–1.17)
PRIMA [32]	III	733	FIGO Stage III with residual tumor IV HGSOC or HGEOC	Niraparib 300 mg once daily, 36 months Placebo once daily, 36 months	Overall population: 13.8 *vs.* 8.2 months	*P* < 0.001 HR: 0.62 (95% CI 0.50–0.76)
					*BRCA*mut: 22.1 *vs*. 10.9 months	HR: 0.40 (95% CI 0.27–.62)
					HRD (including *BRCA*): 21.9 *vs*. 10.4 months	HR: 0.43 (95% CI 0.31–0.59)
					HRD (*BRCA*wt): 19.6 *vs*. 8.2 months	HR: 0.50 (95% CI 0.31–0.83)
					HRP (*BRCA*wt): 8,1 *vs*. 5.4 months	HR: 0.68 (95% CI 0.49–0.94)
VELIA [33]	III	1140	FIGO Stage III/IV HGSOC	Veliparib 150 *BID* in combination, 400 mg *BID* in maintenance	Overall population: 23.5 *vs.* 17.3 months	*P* < 0.001 HR: 0.68 (95% CI 0.56–0.83)
					*BRCA*mut: 34.7 *vs.* 22.0 months	HR: 0.44 (95% CI 0.28–0.68)
					HRD (including *BRCA*): 31.9 *vs.* 20.5 months	HR: 0.57 (95% CI 0.43–0.76)
					HRD (*BRCA*wt): 22.9 *vs.* 19.8 months	HR: 0.74 (95% CI 0.52–1.06)
					HRP (*BRCA*wt): 15.0 *vs.* 11.5 months	HR: 0.81 (95% CI 0.6–1.09)

NR: not reached; wt: wild type

## Conclusion and future perspectives

The recent success of PARP-I in OC represents an unprecedented advance in the therapeutic research for this tumour.

Of note, the evidence that *BRCA* mutated patients derive a great and long-lasting benefit prompted most of the clinicians caring for OC patients to actively search for this mutation occurring in almost 20% of the patients. Since *BRCA* mutation is associated with a significant risk of inherited breast and ovarian cancer, this active search highlighted and in some cases improved the way these patients and mostly their families are cared. The recent data showing some activity also in patients who are proficient in HR or *BRCA* wild type threaten this possibility as the regulatory approval allows to treat all the patients with PARP-I.

Generally, the questions regarding who should be treated and when the PARP-I should be integrated into the OC patients treatment are the two most important issues the clinicians face in deciding the best strategy for these patients.

The answer to these questions is not easy, since none of these trials reports a mature OS analysis that could ultimately help in deciding the best treatment. Moreover, in most of the trials the control arm is represented by chemotherapy alone, whilst the current standard of care is chemotherapy plus bevacizumab (given in combination and as maintenance) and also in the recurrent setting trials most of the patients enrolled were not pretreated with bevacizumab. The only trial in which maintenance with olaparib plus bevacizumab was compared with bevacizumab maintenance alone is PAOLA-1. By design this trial answers to the question of whether maintenance bevacizumab plus olaparib is better than bevacizumab alone. And the data suggest this conclusion. However we lack some important pieces of information on whether PARP-I alone is better than bevacizumab alone (in maintenance) in patients who received bevacizumab in combination with the induction chemotherapy or, based on SOLO-1, at least *BRCA* mutant could derive greater or similar benefit from olaparib alone maintenance instead of the combination olaparib plus bevacizumab, sparing some important toxicity. All these questions deem to be answered in a prospective randomised clinical trial, in our opinion.

Finally, since PARP-I are both approved in recurrent disease and will be approved with various labels in front-line two other questions are arising from the data published up-to-date.

When to administer PARP-I? Front-line or at the recurrence? Is there any role for PARP-I after progression or recurrence while on PARP-I?

The first question is threatened by the lack of OS data for the settings. Also in this case a meaningfully designed clinical trial could answer to this question. The second issue is being explored by the OREO study that is still recruiting (NCT03106987). In this trial, OC patients with the disease relapsing/progressing while on PARP-I and experiencing a complete or partial radiological response to subsequent treatment with platinum-based chemotherapy are randomised to receive olaparib or placebo (2:1).

In conclusion, the advent of PARP-I in OC represents an important resource in the clinical setting for the treatment of patients whose therapy did not greatly change over 20 years and mostly, is still an exciting challenge for the research in this field.

## Abbreviations

AE: adverse event
BER: base excision repair
CR: complete response
EMA: European Medicines Agency
FDA: Food and Drug Administration
gBRCA: germline-BRCA
HGEOC: high-grade endometrioid ovarian cancer
HGSOC: high-grade serous ovarian cancer
HR: homologous recombination
HRD: homologous recombination deficiency
ITT: intention-to-treat
LOH: loss of heterozygosity
MMR: mismatch repair
NER: nucleotide excision repair
NHEJ: non-homologous end joining
OC: ovarian cancer
ORR: overall response rate
OS: overall survival
PARP: Poly-ADP-ribose polymerase
PARP-I: PARP inhibitors
PFS: progression-free survival
PR: partial response
DSB: double-strand break
SSB: single-strand breaks
wt: wild type

## Declarations

### Copyright

© The Author(s) 2020.
